# Peripherin-2 and Rom-1 have opposing effects on rod outer segment targeting of retinitis pigmentosa-linked peripherin-2 mutants

**DOI:** 10.1038/s41598-017-02514-5

**Published:** 2017-05-24

**Authors:** Sybille Böhm, Lisa M. Riedmayr, O. N. Phuong Nguyen, Andreas Gießl, Toni Liebscher, Elisabeth S. Butz, Christian Schön, Stylianos Michalakis, Christian Wahl-Schott, Martin Biel, Elvir Becirovic

**Affiliations:** 1 Center for Integrated Protein Science Munich CiPSM, Ludwig-Maximilians-Universität München, Munich, Germany; 2 Department of Pharmacy – Center for Drug Research, Ludwig-Maximilians-Universität München, Munich, Germany; 3 Department of Biology, Animal Physiology, Friedrich-Alexander Universität Erlangen-Nürnberg, Erlangen, Germany

## Abstract

Mutations in the photoreceptor outer segment (OS) specific peripherin-2 lead to autosomal dominant retinitis pigmentosa (adRP). By contrast, mutations in the peripherin-2 homolog Rom-1 cause digenic RP in combination with certain heterozygous mutations in peripherin-2. The mechanisms underlying the differential role of peripherin-2 and Rom-1 in RP pathophysiology remained elusive so far. Here, focusing on two adRP-linked peripherin-2 mutants, P210L and C214S, we analyzed the binding characteristics, protein assembly, and rod OS targeting of wild type (per^WT^), mutant peripherin-2 (per^MT^), or Rom-1 complexes, which can be formed in patients heterozygous for peripherin-2 mutations. Both mutants are misfolded and lead to decreased binding to per^WT^ and Rom-1. Furthermore, both mutants are preferentially forming non-covalent per^MT^-per^MT^, per^WT^-per^MT^, and Rom-1-per^MT^ dimers. However, only per^WT^-per^MT^, but not per^MT^-per^MT^ or Rom-1-per^MT^ complexes could be targeted to murine rod OS. Our study provides first evidence that non-covalent per^WT^-per^MT^ dimers can be targeted to rod OS. Finally, our study unravels unexpected opposing roles of per^WT^ and Rom-1 in rod OS targeting of adRP-linked peripherin-2 mutants and suggests a new treatment strategy for the affected individuals.

## Introduction

The tetraspanin peripherin-2 is exclusively expressed in outer segments (OS), specific light detecting compartments of photoreceptors. Peripherin-2 forms homo- and heteromeric protein complexes in OS. The core homomeric peripherin-2 unit is the non-covalent tetramer, which can also assemble into covalently linked octamers and higher-order oligomers^1–3^. These homomeric peripherin-2 complexes are crucial for proper OS morphology and architecture^4^. Peripherin-2 also forms heteromeric complexes with its homolog Rom-1, however, the functional importance of these complexes remained unclear^2, 5, 6^. Autosomal dominant retinitis pigmentosa (adRP) is the most frequent hereditary retinal disorder characterized by progressive loss of vision due to rod photoreceptor degeneration. Mutations in the peripherin-2 gene (*PRPH2*) are among the most common causes for adRP^7, 8^. Interestingly, despite the high sequence identity (approx. 35%) and the high structural conservation of Rom-1 and peripherin-2^2, 9^, mutations in Rom-1 are not clearly linked to monogenic adRP. Some homozygous Rom-1 mutations, however, cause digenic RP in combination with a heterozygous mutation in *PRPH2*, suggesting that Rom-1 could function as genetic modifier that shapes the disease progression^10, 11^. Nevertheless, the molecular mechanisms underlying the postulated modifier function of Rom-1 have not been determined yet^12^. The majority of adRP-linked peripherin-2 mutants are located within the large loop domain connecting the transmembrane domain 3 and 4, also known as EC2 or D2 loop domain^13, 14^. Some adRP-linked peripherin-2 mutants in EC2 were shown to influence the subunit assembly^15, 16^. In this context, it was postulated that peripherin-2 tetramerization is crucial for rod OS targeting. However, this conclusion was drawn from experiments addressing pure homotypic per^MT^ complexes^16^. Nevertheless, due to the autosomal dominant fashion of *PRPH2*-linked adRP, different combinations of peripherin-2 protein complexes (per^WT^-per^WT^, per^MT^-per^MT^, per^WT^-per^MT^, Rom-1-per^WT^, and Rom-1-per^MT^) can be formed in heterozygous patients carrying wild type and mutant *PRPH2* alleles. In addition, each of these complexes can exist in different equilibria of mono-, di- and tetramers and higher-order oligomers, respectively. This high complexity precluded a more accurate investigation of how subunit assembly might influence the rod OS targeting of the single peripherin-2 protein complexes.

In previous studies, we provided a proof-of-principle for FRET-based quantitative analysis of protein-protein interactions in isolated rod and cone OS^17–19^. Recently, we also reported that the two adRP-linked mutations (per^P210L^ and per^C214S^) located within the highly conserved tetraspanin PxxCC motif are mislocalized to rod inner segments (IS) when expressed in wild type mice using adeno-associated virus (AAV) vectors^20^. Other studies demonstrated that per^C214S^ affects homotypic mutant interactions and the heteromerization with Rom-1^21, 22^. Despite these findings, little is known about the specific binding characteristics of this and other peripherin-2 mutants to per^WT^ or Rom-1 in their native environment (e.g. rod OS). Moreover, the precise contribution of per^WT^ and Rom-1 on binding properties, subunit assembly, and rod OS targeting of the respective per^WT^-per^MT^ and Rom-1-per^MT^ complexes is not clear. Nevertheless, such information is crucial to understand the full complexity of *PRPH2*-linked adRP and for developing successful treatments for the disease. Here, combining different methods we provide novel insights into the binding characteristics, subunit assembly, and rod OS targeting of per^P210L^ and per^C214S^ mutants. We show that per^WT^ and Rom-1 form heterodimers with both peripherin-2 mutants. However, only per^WT^-per^MT^ but not Rom1-per^MT^ complexes could be targeted to rod OS suggesting unexpected opposing roles of peripherin-2 and Rom-1 in rod OS targeting of adRP-linked peripherin-2 mutants.

## Results

### per^C214S^ and per^P210L^ show reduced binding to per^WT^ and Rom-1

**Figure 1:**
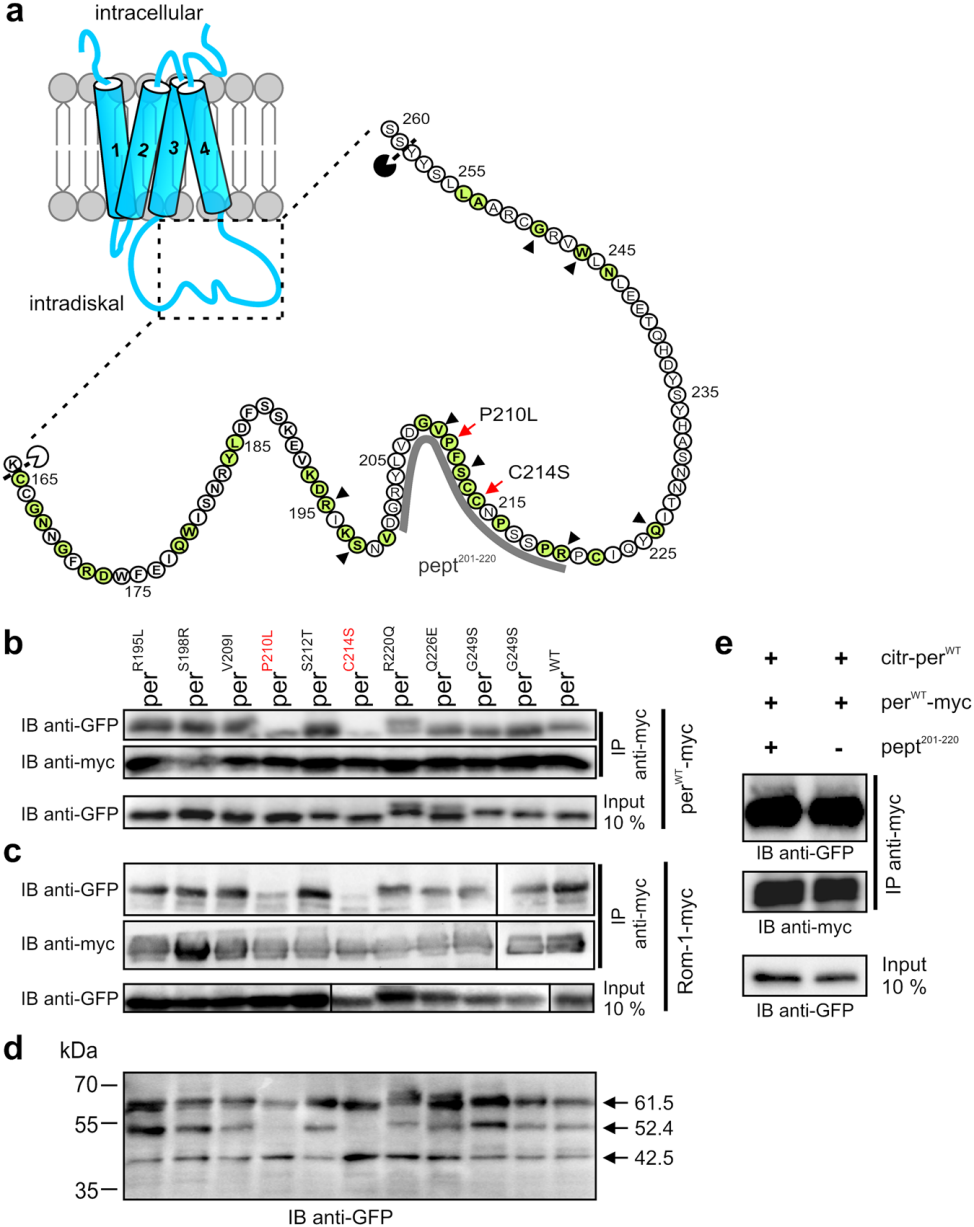
Effects of peripherin-2 mutants on protein-protein interactions and D2 loop folding. (**a**) left, Topology of peripherin-2. The dashed rectangle highlights the distal part of the D2 loop. Right, Schematical enlargement of the distal D2 loop showing the hitherto identified disease-linked peripherin-2 mutations in this region (highlighted in green). The position of mutants analyzed in (**b–d**) are marked by red arrows (for per^P210L^ and per^C214S^) or by black arrowheads in case of the remaining mutants. The calculated protease cleavage sites from d are indicated by a white (corresponding to the 42.5 kDa band) or black (corresponding to the 52.4 kDa band) pacman. The position of the peptide (aa 201–220) used for the competition assay shown in (**e**) (pept^201–220^) is highlighted by the gray line. (**b–c**) Co-Immunoprecipitations (co-IPs) from membrane preparations of HEK293T cells transiently transfected with the respective N-terminally citrine-tagged mutants and C-terminally myc-tagged per^WT^ (per^WT^-myc) (**b**) or Rom-1 (Rom-1-myc) (**c**) using the myc-specific antibody (anti-myc). The single mutants were detected using the GFP-antibody, which also specifically recognizes citrine. IB, immunoblotting. (**d**) Protease cleavage experiments on membrane preparations from HEK293T cells transiently transfected with the single citrine-tagged peripherin-2 mutants as indicated. The molecular weight of the single bands (arrows) was calculated from five independent western blot experiments. (**e**) Co-IPs from membrane preparations of HEK293T cells co-transfected with myc- and citrine-tagged per^WT^ in presence (left lane, 10 mM) or absence of the peptide.

In our recent work, we demonstrated that, in contrast to per^C214S^ and per^P210L^, eight additional disease-linked peripherin-2 D2 loop domain mutants did not affect rod OS targeting^20^ (Fig. 1a). One possible mechanism, which might explain the mislocalization of per^C214S^ and per^P210L^ in rod photoreceptors, is that these mutants might affect binding to per^WT^ and/or to Rom-1. To examine this possibility, we performed co-immunoprecipitation (co-IP) experiments from HEK293T cells co-transfected with the respective peripherin-2 mutants and per^WT^ or Rom-1, respectively. Only per^C214S^ and per^P210L^ attenuated the binding to per^WT^, while binding of other peripherin-2 mutants to per^WT^ remained unaffected (Fig. 1b). Very similar results were obtained in co-IPs addressing the binding of the single peripherin-2 mutants to Rom-1 (Fig. 1c).

### P210 and C214 are crucial for the proper folding of the distal D2 loop

The proline at position 210 (P210) and the cysteine at position 214 (C214) are part of the highly conserved tetraspanin PxxCC motif, whose function has not been clearly determined yet. The reduced binding of per^C214S^ and per^P210L^ to per^WT^ suggests that these residues could either i) be crucial for proper folding of peripherin-2 or ii) represent the protein-protein interaction interface. Using CD spectroscopy, a recent study suggested that C214S impedes protein folding by increasing the percentage of ß-sheets concomitant with a decrease in helices in the D2 loop^23^. However, it remained unclear which part(s) of the D2 loop is (are) affected by this mutation. To address this issue more directly, we established a protease digestion assay on membrane preparations from HEK293T cells expressing citrine-tagged WT and mutant peripherin-2 transgenes. For this purpose, we took advantage of the fact that endogenously expressed proteases are regularly present in these preparations. In addition, membrane preparations are expected to retain the native protein structure as they are isolated without detergents or reducing agents. Protein cleavage occurs on the exposed domain regions, which are accessible for endogenous proteases. Accordingly, improper protein folding could lead to masking of existing or to an exposure of hidden protease cleavage sites. Using a GFP antibody that recognizes the N-terminal citrine tag of our peripherin-2 constructs, we could detect three peripherin-2 bands at 61.5 kDa, 52.4 kDa and 42.5 kDa (Fig. 1d). The calculated size of full-length citrine-tagged peripherin-2 is 66.5 kDa, however, even in presence of protease inhibitors, the full-length per^WT^ was always detected as a 61.5 kDa band. By contrast, the 52.4 kDa and 42.5 kDa could only be detected in the protease cleavage assay in the absence of protease inhibitors and upon incubation of the samples at 37 °C. Using the 61.5 kDa band as reference, we calculated the cleavage sites for the 42.5 kDa and for the 52.4 kDa band. The cleavage site for the 42.5 kDa band corresponds to the peripherin-2 region around the highly conserved tetraspanin CCG motif (Fig. 1a). This band was also present in all peripherin-2 mutants suggesting proper folding at this part of the protein. By contrast, the 52.4 kDa band was completely absent for per^C214S^ and per^P210L^, whereas other mutations did not show any considerable differences to per^WT^. The protease cleavage site for the 52.4 kDa band is calculated to be located within the distal part of the D2 loop domain next to the transmembrane domain 4 (Fig. 1a). Hence, these results suggest that per^C214S^ and per^P210L^ lead to a structural rearrangement of the distal part of the D2 loop domain.

A recent study suggested that peripherin-2 residues 165–182 are crucial for homomeric interactions^24^. However, it remained unclear whether the per^C214S^ and per^P210L^ residues or flanking regions might also contribute to this type of interactions. To test this possibility, we performed a peptide competition assay using a peptide corresponding to the residues 201–220 of native human peripherin-2. Provided that the positions P210 and C214 participate in peripherin-2 homomerization, this peptide should compete with the binding and, hence, result in a reduction of homomeric protein-protein interactions. However, in subsequent co-IP experiments even in the presence of a very high peptide concentration (10 mM), we could not detect any decrease in peripherin-2 binding compared to the peptide-free approach (Fig. 1e). This suggests that this region is not directly involved in homomeric peripherin-2 protein-protein interactions.

### Quantification of per^WT^, per^MT^, and Rom-1 protein-protein interactions in HEK293T cells using FRET

**Figure 2:**
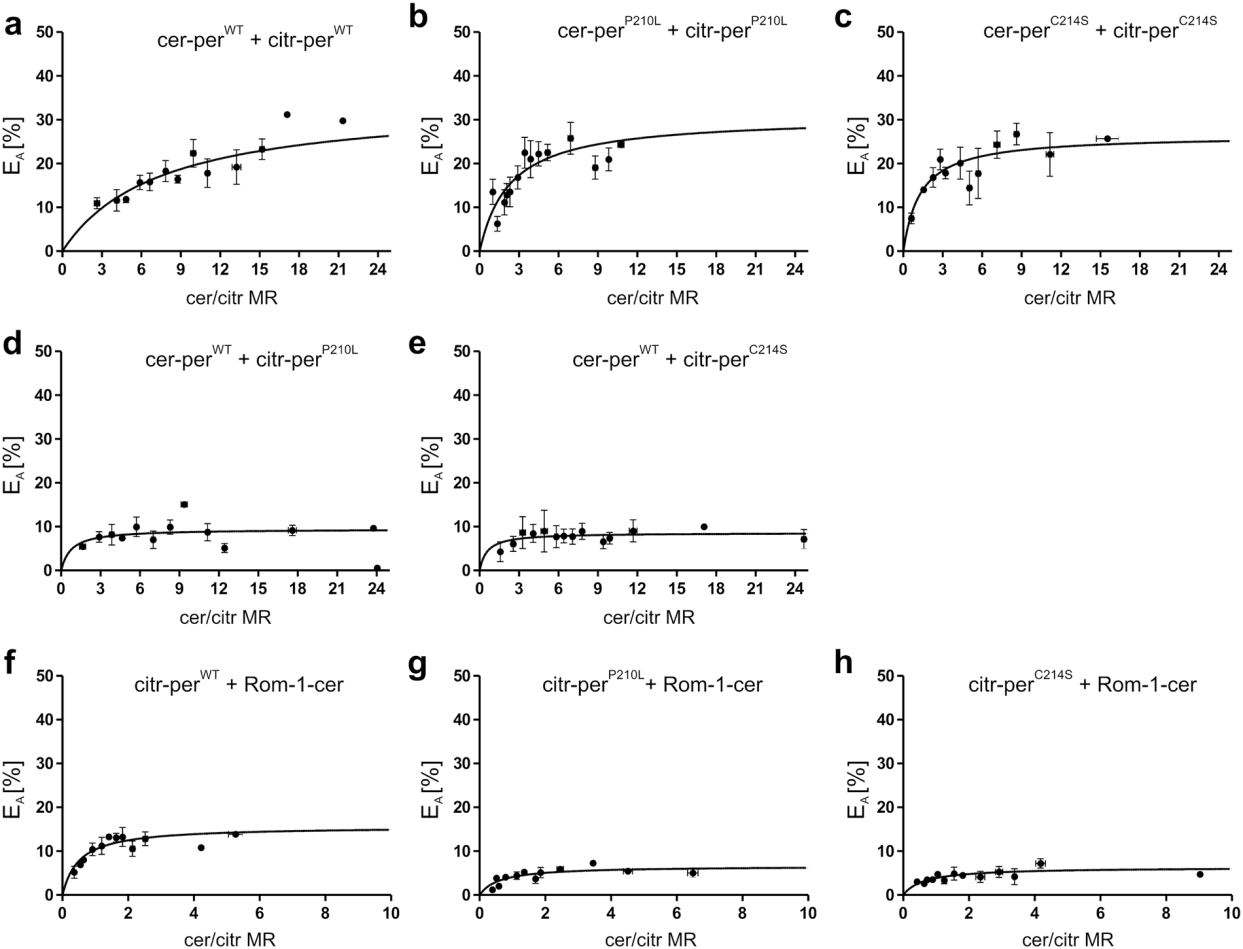
Quantification of homo- and heteromeric per^WT^, per^MT^, and Rom-1 protein-protein interactions using FRET. (**a–h**) FRET experiments from living HEK293T cells transiently co-transfected with the respective citrine- (citr) or cerulean (cer)-tagged peripherin-2 or Rom-1 constructs as indicated. To obtain the binding curves, FRET efficiency (E_A_) was plotted against the cerulean/citrine molar ratio (cer/citr MR). The dots represent mean values of 1–7 single FRET measurements ± SEM. E_Amax_, maximal FRET efficiency. The single E_Amax_, values and the numbers of independent measurements (n) for each combination are summarized in Supplementary Table 1.

Recently, we reported that three cube FRET is suitable for quantification of homomeric and heteromeric interactions of different photoreceptor specific proteins including peripherin-2. In particular, it has been demonstrated that FRET can be used to calculate the relative binding affinities of these proteins in HEK293T cells^17^. Consequently, using this approach we examined the binding properties of the different homo- and heteromeric per^WT^, per^MT^, and Rom-1 protein complexes. Binding curves can be calculated if the relative expression of the FRET fluorophores (given as molar ratio, MR) varies between the single FRET measurements. By plotting the FRET efficiency (E_A_) values against the corresponding cerulean/citrine MR, we observed a sufficient variability of the FRET fluorophore expression for each combination. The maximal FRET efficiency (E_Amax_) was calculated from the binding curve for saturable binding as described previously^17^ and is proportional to the binding strength of the respective protein complex in the equilibrium. Analysis of E_Amax_ for the different per^WT^ and per^MT^ combinations allowed us to draw several important conclusions. First, robust and unusually high E_Amax_ values could be observed for the per^WT^ only FRET pair (34.2%, Fig. 2a and Supplementary Table 1). These values are close to the highest theoretically possible 40% FRET efficiency for proteins tagged with bulky fluorophores like citrine or cerulean and highlight the strength of protein-protein interactions in homomeric peripherin-2 complexes. Second, E_Amax_ was only slightly decreased for the per^P210L^ only (30.7%) and per^C214S^ only (26.47%) FRET pairs when compared to per^WT^ only (Fig. 2b,c and Supplementary Table 1), suggesting that mutants are still largely capable of self-interacting. By contrast, both, per^WT^-per^P210L^ (9.44%) and the per^WT^-per^C214S^ (8.58%) combinations, led to a robust decrease in E_Amax_ (Fig. 2d,e and Supplementary Table 1). Next, we also determined the E_Amax_ values for Rom-1 co-expressed with each, per^WT^ and per^MT^. When compared to per^WT^ only, E_Amax_ for the Rom-1-per^WT^ interaction was noticeably lower (15.64%) indicating that heteromeric Rom-1-per^WT^ complexes bind less tight than their homomeric per^WT^ only counterparts (Fig. 2f and Supplementary Table 1). Second, both, Rom-1-per^P210L^ and Rom-1-per^C214S^ combinations, led to reduced E_Amax_ values when compared to the Rom-1-per^WT^ interaction (6.58% and 6.32%, respectively, Fig. 2g,h and Supplementary Table 1). Finally, we also calculated the percentage of E_Amax_ reduction for both peripherin-2 mutants relative to per^WT^ only and to the per^WT^-Rom-1 FRET pair. When compared to per^WT^ only, the E_Amax_ values were reduced to 28% for the per^WT^-per^P210L^ and to 25% for the per^WT^-per^C214S^ FRET pair, respectively. By contrast, relative to the Rom-1-per^WT^ FRET pair, the E_Amax_ values were reduced to 42% for the Rom-1-per^P210L^ and to 40% for the Rom-1-per^C214S^ combination, respectively (Supplementary Table 1). This suggests that binding of both peripherin-2 mutants to per^WT^ is more strongly affected than binding to Rom-1.

Taken together, our FRET results indicate that, although with reduced efficiencies, both mutants in principle retain their ability to self-interact and to form complexes with per^WT^ or with Rom-1.

### Subunit assembly of homo- and heteromeric per^WT^, per^MT^, and Rom-1 complexes

**Figure 3:**
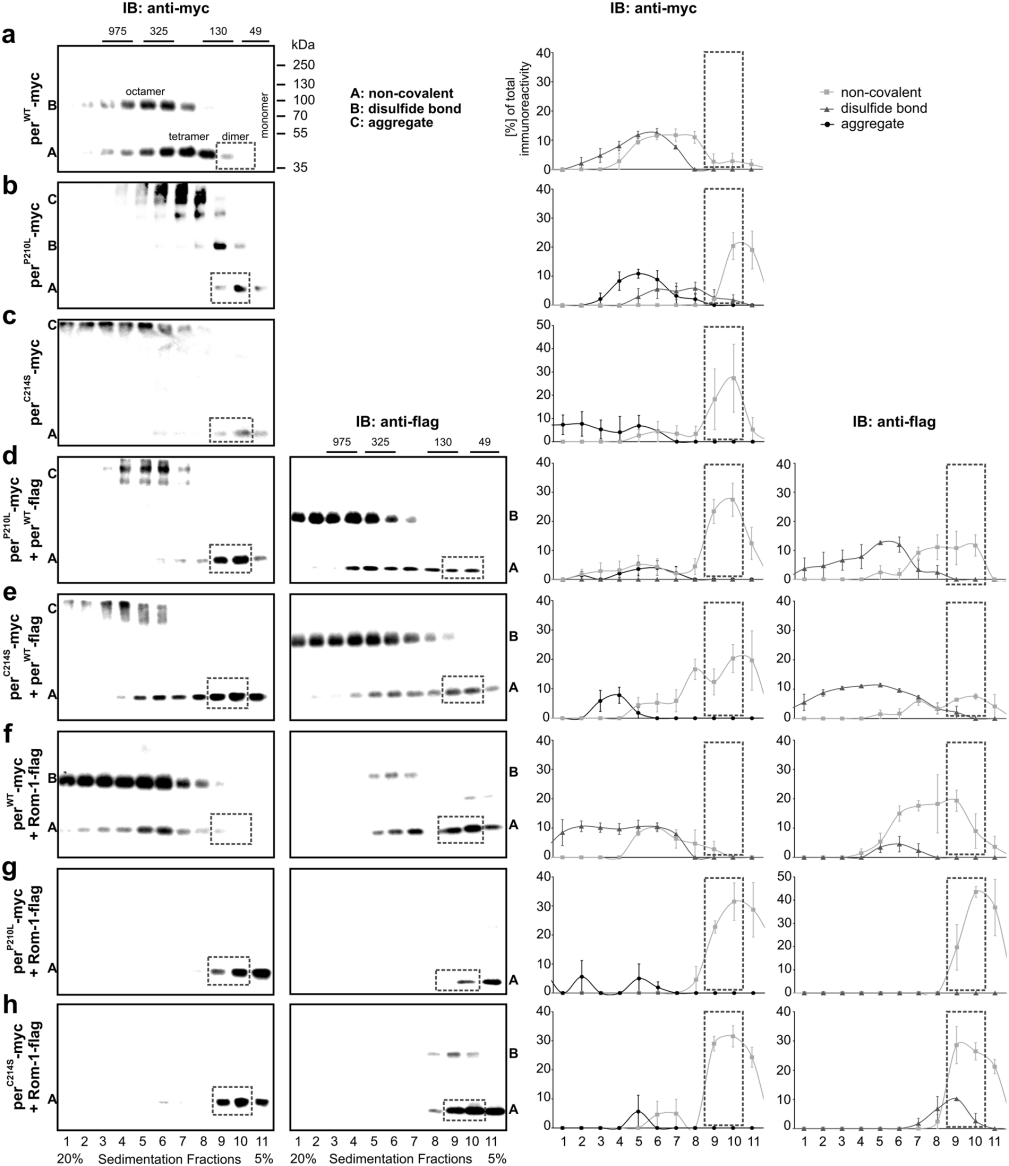
SDGC of different per^WT^, per^MT^, and Rom-1 combinations. Shown are representative immunoblots (left panel) and the corresponding statistics (right panel) for each C-terminally myc- or flag-tagged combination as indicated. Each blot consists of 11 fractions collected across the 5–20% sucrose gradient. All fractions were normalized to the DNA standard mixed to the protein solution prior to SDGC as described in the methods section. The single peripherin-2 or Rom-1 complexes can be found in following fractions: octamers: fraction 3–6; tetramers: fraction 6–8; dimers: fraction 9–10; monomers: fraction 10–11. Immunoblottings were performed with antibodies recognizing either the myc-tag (first row of the left panel) or the flag-tag (second row of the left panel), respectively. For quantification, the percentage of total immunoreactivity related to the number of single fractions for non-covalent or covalent complexes and aggregates was plotted against the 11 collected fractions. The fractions containing the non-covalent dimers are highlighted by a dashed rectangle. The single values (percentages and numbers of independent experiments) for all combinations are summarized in Supplementary Table 2. Fractions containing the DNA standards (975 kDa, 325 kDa, 130 kDa and 49 kDa) are marked accordingly.

Sucrose density gradient centrifugation (SDGC) allows for a separation of different covalently or non-covalently linked protein complexes across a sucrose gradient according to their specific molecular weight. Using SDGC, previous studies analyzed the impact of peripherin-2 mutants including per^C214S^ on homotypic per^MT^-per^MT^ or heterotypic Rom-1-per^MT^ interactions on subunit assembly^16, 21, 22, 25^. To examine the complexes which can be formed in heterozygous patients more systematically, we performed a set of sedimentation velocity experiments of differentially tagged per^WT^, per^MT^, and Rom-1 combinations (per^WT^ only, per^P210L^ only, per^C214S^ only, per^WT^-per^P210L^, per^WT^-per^C214S^, Rom-1-per^WT^, Rom-1-per^P210L^, and Rom-1-per^C214S^) transiently expressed in HEK293T cells. In line with previous work, we found the per^WT^ complexes only in sucrose gradient fractions containing the non-covalent tetramers as well as in fractions containing the disulfide-linked octamers under non-reducing conditions (Fig. 3a). By contrast, when expressed alone, per^P210L^ was primarily detected as monomer, non-covalent dimer, and aggregates, but some complexes were also found as disulfide-linked tetramers and octamers (Fig. 3b). Similar to per^P210L^, per^C214S^ was predominantly detected in monomer, non-covalent dimer, and aggregate fractions when expressed alone (Fig. 3c). In presence of per^WT^, per^P210L^ was primarily detected in the fractions containing monomers and non-covalent dimers, and only weak signal was found in the non-covalent tetramer fractions (Fig. 3d). Moreover, in this combination the SDGC pattern for per^WT^ slightly differed from the per^WT^ only situation. The most striking difference was that a substantial per^WT^ signal could now also be seen in the non-covalent dimer fractions (Fig. 3d). Given that no considerable amounts of per^WT^ were detectable in this fraction for per^WT^ only (cf. Fig. 3a), we concluded that the non-covalent dimer fraction most likely consists of per^WT^-per^P210L^ dimers. When co-expressed with per^C214S^, per^WT^ reasonably decreased the percentage of per^C214S^ aggregates (Fig. 3e). Moreover, in presence of per^WT^ the majority of per^C214S^ was found in the non-covalent dimer fractions and as per^C214S^ monomers. However, in contrast to the per^WT^-per^P210L^ combination, a strong per^C214S^ signal could also be detected in the non-covalent tetramer fractions. Finally, in the per^WT^-per^C214S^ combination, we could also robustly detect per^WT^ in the non-covalent dimer fractions (Fig. 3e). Taking into account that per^WT^ alone does not homodimerize (cf. Fig. 3a) and that per^C214S^ alone does not form non-covalent tetramers (cf. Fig. 3c), we concluded that the non-covalent dimer and tetramer fractions in the per^WT^-per^C214S^ combination most likely contain per^WT^-per^C214S^ dimers and tetramers, respectively (Fig. 3e).

Next, we analyzed the SDGC fractions from HEK293T cells co-expressing Rom-1-per^WT^, Rom-1-per^P210L^, and Rom-1-per^C214S^ complexes. As expected, in the Rom-1-per^WT^ combination a robust Rom-1 signal was found in non-covalent tetramer fractions (Fig. 3f). In addition, Rom-1 was also detected in non-covalent dimer and monomer fractions. No considerable amounts of per^WT^ were found in these fractions suggesting that no Rom-1-per^WT^ heterodimers are present under these conditions (Fig. 3f). When analyzing Rom-1-per^P210L^ and Rom-1-per^C214S^ combinations, the per^P210L^ and per^C214S^ mutants were almost exclusively found in the non-covalent dimer and monomer fractions (Fig. 3g,h). In contrast to the Rom-1 signal from the Rom-1-per^WT^ combination, in presence of the per^P210L^ and per^C214S^ mutants Rom-1 was almost exclusively detected in dimer and monomer fractions (Fig. 3g,h). Based on this, we concluded that Rom-1 predominantly forms non-covalent dimers with both mutants.

Taken together, our SDGC results suggest that per^WT^-per^MT^ and Rom-1-per^MT^ protein complexes are mainly built of non-covalent dimers.

### Rod OS targeting of homo- and heteromeric per^WT^, per^MT^, and Rom-1 complexes

**Figure 4:**
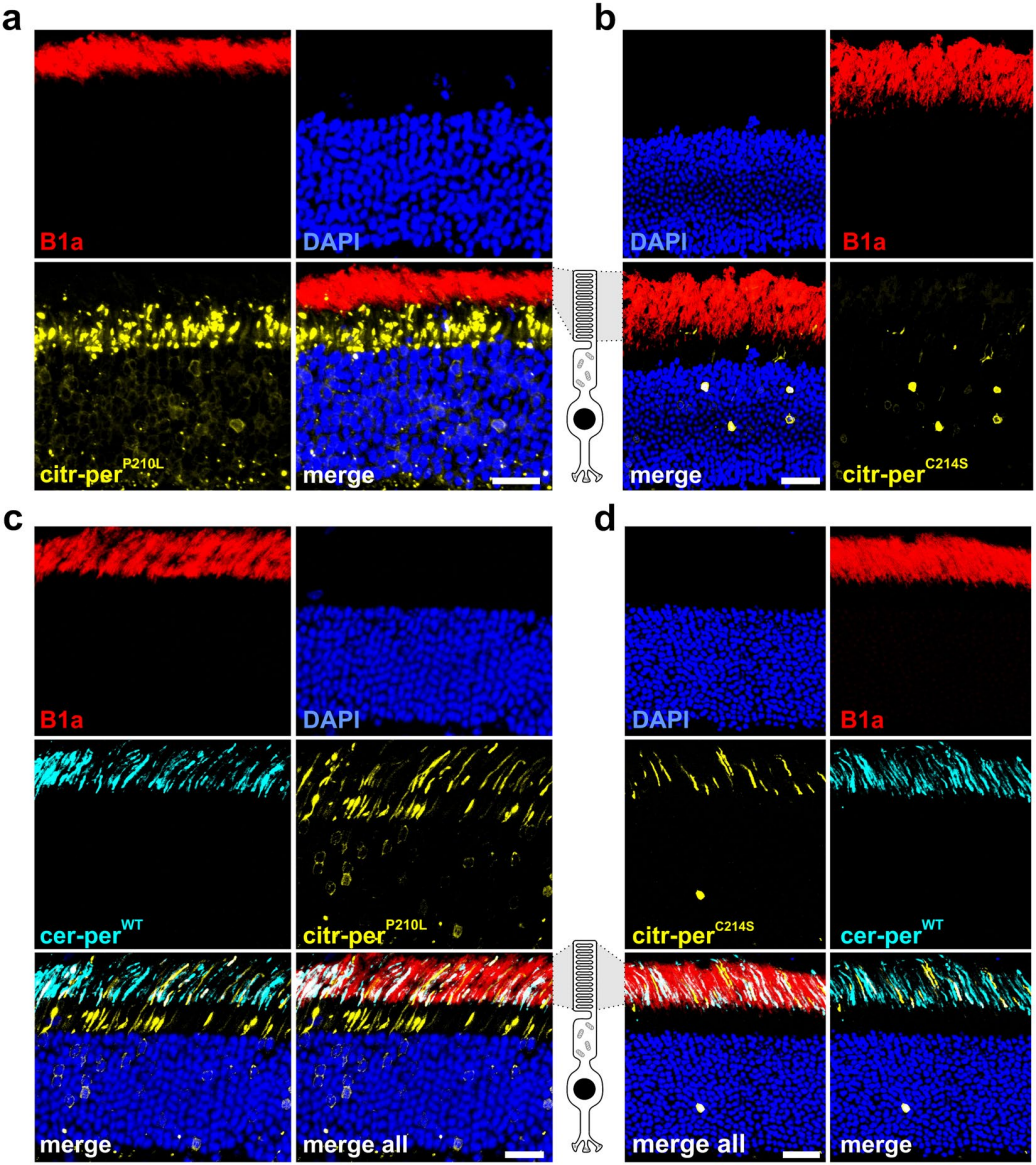
Rod OS targeting of per^WT^-per^MT^ complexes. (**a–d**) Confocal images of immunostained retinas injected with citrine-tagged mutants (citr-per^P210L^ (**a**) and citr-per^C214S^ (**b**)), or co-injected with the respective mutants and the N-terminally cerulean-tagged per^WT^ (cer-per^WT^, (**c–d**)). CNGB1a antibody (B1a, red) was used for specific staining of rod OS. Scale bar, 30 µm.

**Figure 5:**
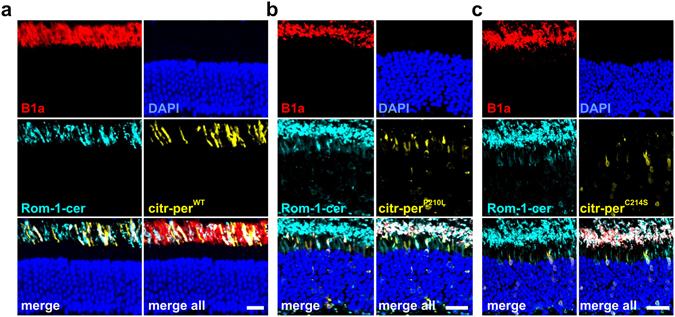
Rod OS targeting of Rom-1-per^WT/MT^ complexes. Confocal images of immunostained retinas co-injected with C-terminally cerulean-tagged Rom-1 (Rom-1-cer) and citr-per^WT^ (**a**), citr-per^P210L^ (**b**), and citr-per^C214S^ (**c**). Scale bar, 30 µm.

Our FRET and SDGC experiments from HEK293T cells strongly suggest that both peripherin-2 mutants are in principle capable of forming complexes with per^WT^ and Rom-1. This prompted us to analyze the protein targeting of these complexes in rod photoreceptors. For this purpose, we used rAAV vector gene delivery to express various combinations of per^WT^, Rom-1, and per^MT^ and analyze their transport/localization in mouse photoreceptors. It has been shown previously that subretinal co-administration of two rAAVs encoding different transgenes results in very high co-transduction efficiencies in rods^17^. For delivery to rod photoreceptors, rAAV vectors with a rod specific human rhodopsin promoter were used. To allow for discrimination between per^WT^ or Rom-1 and per^MT^ expression, we fused cerulean to the N-terminus of per^WT^ and Rom-1, and citrine to the N-terminus of per^MT^, respectively. The rAAVs containing the different peripherin-2 and Rom-1 constructs were subretinally injected to P14 wild type mice. Subsequent analysis of peripherin-2 localization was examined four weeks post injection by detecting citrine and cerulean fluorescence on retinal slices from injected animals. When expressed alone, citrine and cerulean were retained in the rod IS (Suppl. Fig. 1). In accordance with our previous observations^20^, both mutants were also completely mislocalized to the IS (Fig. 4a,b). The localization pattern of per^P210L^ and per^C214S^ was remarkably different. per^P210L^ showed a spotted large vesicular-like expression, whereas per^C214S^ was distributed throughout the rod IS. Interestingly, however, simultaneous co-delivery of titer-matched per^WT^ and per^MT^ led to a noticeable rescue and substantial rod OS targeting of per^P210L^ and per^C214S^ (Fig. 4c,d). In addition, the rAAV-mediated co-delivery of per^WT^ also rescued the vesicle-like expression pattern of per^P210L^ in the rod IS (Fig. 4c). Finally, we also assessed whether co-administration of titer-matched rAAVs expressing transgenic Rom-1 together with per^P210L^ or per^C214S^ leads to a similar rescue effect. When co-expressed with per^WT^, both, Rom-1 and per^WT^ were exclusively found in rod OS (Fig. 5a). However, transgenic Rom-1 did not noticeably promote per^MT^ protein targeting to the OS. By contrast, a substantial amount of transgenic Rom-1, most likely the Rom-1-per^MT^ heterodimers, was retained in the rod IS (Fig. 5b,c).

Taken together, these experiments demonstrate that simultaneous co-delivery of transgenic per^WT^ rescues the targeting of the per^P210L^ and per^C214S^ mutants to rod OS. However, simultaneous co-delivery of transgenic Rom-1 and per^P210L^ or per^C214S^ resulted in substantial retention of Rom-1 in the rod IS. In combination with the results obtained from SDGC experiments, these findings strongly indicate that per^WT^-per^MT^, but not Rom-1-per^MT^ heterodimers, can be efficiently targeted to rod OS.

### Analysis of the per^WT^-per^MT^ binding properties in rod OS using FRET

**Figure 6:**
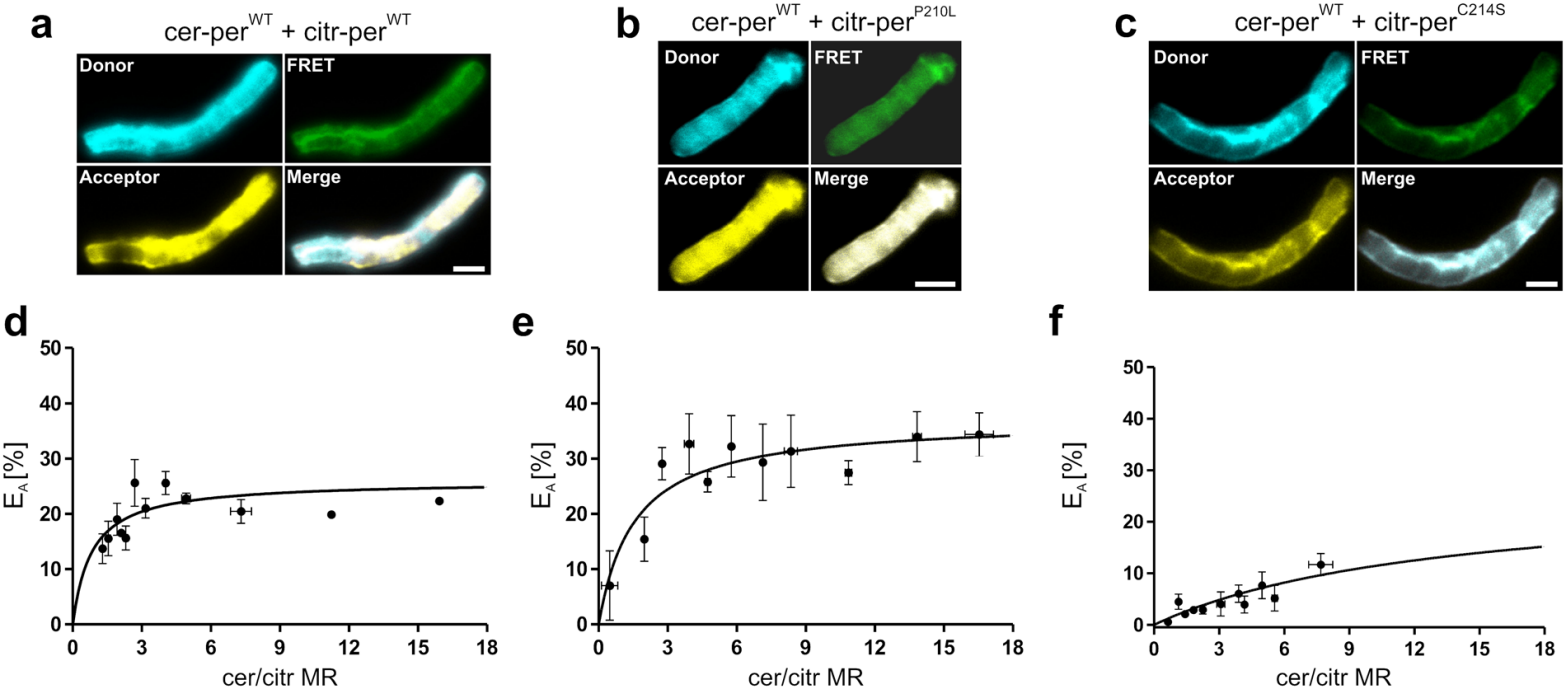
FRET experiments in rod OS of retinas co-injected with per^WT^ and per^MT^ AAV-vectors. (**a–c**) Representative confocal images of FRET channels showing a single OS co-expressing cer-per^WT^ and citr-per^WT^(**a**), cer-per^WT^ and citr-per^P210L^ (**b**), or cer-per^WT^ and citr-per^C214S^ (**c**). Scale bar, 2 µm. (**d–f**) Binding curves of the corresponding FRET measurements for the single combinations displayed in (**a–c**). The dots represent mean values of 1–7 single FRET measurements ± SEM. The single E_Amax_, values and the numbers of independent measurements (n) for each combination are summarized in Supplementary Table 1.

FRET and co-IP experiments in HEK293T cells revealed a reduced apparent binding of both peripherin-2 mutants to per^WT^. This finding could be best explained by decreased binding affinities of the per^WT^-per^MT^ interaction and/or decreased initial binding kinetics of the mutants to per^WT^. However, in HEK293T cells co-transfected with per^WT^ and per^MT^ each mutant can also exist as monomers or could undergo self-interactions (i.e. as dimers or aggregates, cf. Fig. 3b–e). As all these combinations would contribute to the overall FRET signal, the apparent reduction of E_Amax_ in HEK293T cells does not allow for discrimination between the mechanisms described above. To examine this issue directly, we took advantage of the fact that per^MT^ targeting to rod OS is rescued only upon co-delivery of transgenic per^WT^. Consequently, in contrast to HEK293T cells, in rescued rod OS all per^MT^ protein must be arranged in complexes with per^WT^. This happenstance allowed us to draw direct conclusions about the per^WT^-per^MT^ binding properties under close-to-native conditions by measuring FRET in isolated “rescued” rod OS. For this purpose, we co-injected wild type mice on P14 with the respective per^WT^ and per^MT^ FRET pairs. At four weeks post injection we isolated the rod OS and measured FRET on single OS co-expressing both, cerulean-tagged per^WT^ and citrine-tagged per^MT^ (Fig. 6a–c). Robust FRET signals could be measured for all combinations. Importantly, the cerulean/citrine molar ratios substantially varied between the single rod OS, which also allowed us to calculate the binding curves. Remarkably, in contrast to the FRET results from HEK293T cells, the E_Amax_ from isolated rod OS was either increased in case of the per^WT^-per^P210L^ pair or very close to that of the per^WT^ only interaction for the per^WT^-per^C214S^ combination (Fig. 6d–f and Supplementary Table 1). These findings indicate that the robust reduction in the per^WT^-per^MT^ interaction observed from co-IP and FRET experiments in HEK293T cells does not result from reduced binding affinity, but is most likely caused by a decrease in initial binding kinetics of per^MT^ to per^WT^.

## Discussion

By analyzing the molecular mechanisms of two adRP-linked peripherin-2 mutants, we provide evidence for a novel role of peripherin-2 and its homolog Rom-1 in the pathophysiology of rod photoreceptors. We show for the first time that rod OS targeting of peripherin-2 mutants can be rescued *in vivo* by simultaneous co-delivery of per^WT^ using rAAV-mediated gene transfer. This finding is important for understanding the peripherin-2 biology in photoreceptors and has implications for the development of future gene therapies for patients with *PRPH2* mutations. Peripherin-2 is crucial for proper rod OS structure and development. This function was proposed to be dose-dependent in rods suggesting that reduced dosage of peripherin-2 (less than 60–80% of the wild type level) induces retinal degeneration^26, 27^. In line with the postulated dose-dependency, transgenic mice expressing the per^C214S^ mutant show reduced amounts of mutant protein in rod OS (due to mislocalization or protein degradation) which results in haploinsufficiency^22^. Interestingly, genetic supplementation of per^WT^ on the per^C214S^ mutant background led to a noticeable rescue of the retinal function^28^. Although helpful in initial stages of therapy development, the application spectrum of genetic supplementation is restricted to model organisms. By contrast, rAAV-mediated gene delivery has proven to be a very promising technique for therapeutic intervention on retinal diseases like retinitis pigmentosa in mammals^29–32^. Our data suggest that rAAV-mediated gene supplementation of per^WT^ could support a rescue of per^MT^ targeting defects to rod OS in adRP patients carrying mislocalizing peripherin-2 mutations. We could not detect any detrimental effects on outer nuclear layer (ONL) thickness in fully developed retinas of WT animals co-injected with per^WT^ and per^MT^ transgenes six months post injection (Suppl. Fig. 2). However, we cannot exclude the possibility that rescue of per^MT^ rod OS targeting by per^WT^ gene supplementation would initiate retinal degeneration in patients heterozygous for these mutations. Consequently, prior to gene therapy, the long-term effects of the mutant rescue on retinal degeneration should be tested in appropriate animal models.

In mice co-injected with rAAVs expressing labeled peripherin-2 and Rom-1 transgenes, it is not possible to estimate the contribution of the endogenous murine peripherin-2 and Rom-1 on the per^MT^ rescue. However, our observation that both mutants are mislocalized to the rod IS and lead to reduced protein expression suggests that the endogenous protein levels were not sufficient to rescue per^MT^ localization. Of note, in heterozygous transgenic knock-in mice expressing per^C214S^, the mutant protein remains in the inner segments and is highly prone to degradation^22^. These results are very similar to our observations from wild type mice injected with the transgenic per^C214S^ mutant. With respect to this finding, it can be concluded that the rAAV-mediated delivery of transgenic per^C214S^ mutant to wild type mice largely reflects the native situation in mice heterozygous for this mutant. The fact that per^C214S^ is not targeted to rod OS suggests that per^WT^-per^C214S^ complexes most likely are not formed under these conditions. However, as discussed above, additional rAAV-mediated delivery of per^WT^ presumably exceeds the critical levels necessary for per^WT^-per^MT^ complex formation, highlighting the critical role of peripherin-2 dosage in pathophysiology of photoreceptors.

Our study also provides novel insights into the structural impacts of per^P210L^ and per^C214S^ on folding of the D2 loop domain. We show that per^P210L^ and per^C214S^ lead to a structural rearrangement of the distal part of the D2 loop domain around the aa position 260. It is conceivable that per^P210L^ and per^C214S^ may also lead to structural changes in other parts of the D2 loop, which could not be covered by our assay. However, our data suggest that folding changes induced by per^P210L^ and per^C214S^ most likely do not elicit structural rearrangements of the proximal half of the D2 loop close to the highly conserved tetraspanin CCG motif.

P210 and C214 are located within the PxxCC motif, which is conserved among most tetraspanins^33^. While both cysteine residues of the motif were shown to be involved in intramolecular peripherin-2 disulfide bond formation^21, 34^, the role of the proline residue remained unknown. Peripherin-2 belongs to the subfamily of non-conventional tetraspanins^34^, thus, it remains to be clarified if the PxxCC motif has a similar role on protein folding in other tetraspanins. Interestingly, other mutants in close proximity to the P210 and C214 position (i.e. per^V209I^ or per^S212T^) did not change the accessibility of the proteases in our assay. This emphasizes the crucial role of the proline at position 210 and cysteine at position 214 for the proper folding of the D2 loop domain. Furthermore, our results suggest that the apparent reduction of per^MT^ binding to per^WT^ in co-IP and in FRET experiments from HEK293T cells is most likely caused by protein misfolding. Our FRET experiments on isolated rod OS indicate that this apparent decrease in binding to per^WT^ does not result from reduced binding affinities, but rather seems to be a consequence of reduced initial binding kinetics of per^P210L^ and per^C214S^ to per^WT^. In other words, this suggests that the first step of per^MT^ binding to per^WT^ is impeded, however, once bound, the affinity of both mutants to per^WT^ remains largely unchanged. These results are well matched on our peptide competition assay indicating that the PxxCC motif does not represent the interface for homomeric peripherin-2 interactions. E_Amax_ values resulting from the transgenic per^WT^ were higher in HEK293T cells compared to the rod OS. This difference could be caused by non-labeled endogenous peripherin-2 or Rom-1 in the retina, which are also expected to bind to transgenic per^WT^ (incomplete labeling). However, the per^WT^ E_Amax_ values from isolated rod OS are still in a very high range highlighting the suitability of the FRET approach to addressing peripherin-2 oligomerization in these compartments.

**Figure 7:**
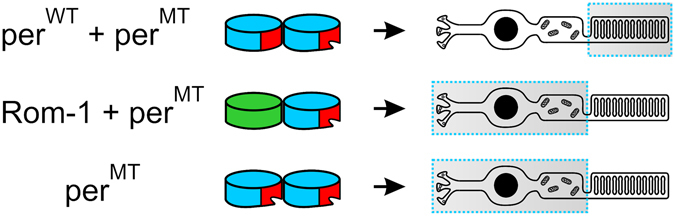
Rod OS targeting of per^MT^ dimeric complexes. The rod photoreceptor localization of different per^MT^-containing dimers (per^WT^-per^MT^, Rom-1-per^MT^ and per^MT^ only) is symbolized by a dashed rectangle. The position of the D2 loop is highlighted in red and the D2 loop misfolding caused by per^MT^ is symbolized by an incision.

When correlating our results from SDGC to those obtained from rod OS targeting experiments (Fig. 7 and Suppl. Fig. 3), two very important conclusions could be drawn: 1) Non-covalent per^WT^-per^MT^ dimers can be formed and targeted to rod OS. 2) Non-covalent Rom-1-per^MT^ heterodimers are stacked in the inner segments. The first deduction is very surprising as non-covalent tetramerization was considered to be crucial for peripherin-2 targeting to rods^16^. This conclusion was drawn from different sets of experiments in previous studies: i) When analyzing per^WT^ complexes isolated from OS, no dimers could be detected^35^. ii) Tetramerization-deficient peripherin-2 mutants could not be targeted to rod OS^16^. iii) Peripherin-2 mutants which preferentially form homodimers are not targeted to rod OS^16^. However, none of these experiments is sufficient to state if per^WT^-per^WT^ or per^WT^-per^MT^ dimers can also be targeted to rod OS. First, per^WT^ does not form dimers in SDGC experiments when expressed alone, which explains its absence in rod OS under physiological conditions. Second, only rod OS targeting of pure per^MT^ complexes was examined so far^16^. Consequently, to the best of our knowledge, this is the first study addressing the principle capability of per^WT^-per^MT^ dimers to be targeted to rod OS. The finding that Rom-1-perMT heterodimers cannot be targeted to rod OS suggests that Rom-1 cannot fully compensate for the peripherin-2 function in rods under these conditions. Currently, one can only speculate about the physiological role of these opposing effects of peripherin-2 and Rom-1 in terms of binding and targeting of misfolded peripherin-2 mutants. A recent study revealed that Rom-1 promotes the trafficking of the Y141C peripherin-2 mutant *in vitro* and converts the cone-dominant pattern dystrophy to RP phenotype *in vivo* ^12^. Similar effects are not expected to occur for the C214S or P210L mutation, as Rom-1 cannot promote targeting of these mutants to rod OS. Together, these findings indicate that Rom-1 might play a yet undefined role in pathophysiology of retinal diseases as a mutation-dependent disease modifier.

Rom-1 and peripherin-2 are also expressed in cones and many mutations in peripherin-2 (but not in Rom-1) are linked to retinal diseases primarily affecting cone photoreceptors^13^. Despite some advances, the molecular mechanisms underpinning this differential penetrance of peripherin-2 mutants in rods and cones are still not fully understood. In light of our results, it is tempting to speculate that Rom-1 might be one of the key players shaping the penetrance of peripherin-2 mutants in rods and cones. Finally, in the context of gene therapy, the postulated novel function of Rom-1 raises the possibility that the efficiency of gene supplementation therapy of adRP-associated peripherin-2 point mutations could be improved by co-delivering peripherin-2 with Rom-1.

## Methods

### Animals

The studies were carried out in accordance with the approved guidelines of the local committee of laboratory animal care (District Government of Upper Bavaria) and German Laws on animal welfare (Tierschutzgesetz). Anesthesia was performed by intraperitoneal injection of ketamine (40 mg/kg body weight) and xylazine (20 mg/kg body weight). Euthanasia was performed by cervical dislocation.

### Molecular biology

For expression in rods, the single human peripherin-2^20^ and Rom-1 transgenes were subcloned to the pAAV2/8 YF vector^30^ containing the human Rhodopsin (hRHO) promoter using standard cloning techniques. For expression in HEK293T cells (LentiX 293 T Cell Line, Clontech Laboratories, Mountain View, CA, USA), the peripherin-2 and Rom-1 transgenes were subcloned to the standard pcDNA3.1 vector (Invitrogen, Waltham, MA, USA). Peripherin-2 mutants were introduced by site directed mutagenesis (QuikChange Lightning Site-Directed Mutagenesis Kit, Agilent Technologies, Santa Clara, CA, USA). All transgenes were sequenced prior to use.

### Protein biochemistry

For co-immunoprecipitations (co-IPs) transiently transfected HEK293T cells were harvested 48 hours post transfection and membrane preparations were conducted as described^36^. Co-IPs were performed with the anti-myc antibody (9B11, Cell Signaling Technology, Danvers, MA, USA) using Protein G magnetic beads (Novex, Thermo Fisher Scientific, Waltham, MA, USA) according to the manufacturer’s protocol.

For western blotting, injected retinas of light adapted wild type C57BL6/J animals were used four weeks post injection. The dissected retinas were homogenized in membrane preparation buffer using the Potter S homogenizer (B. Braun Diessel Biotech, Melsungen, Germany).

For peptide competition assay, the peripherin-2 peptide (peptide sequence CDGRYLVDGVPFSCCNPSSPR) was obtained from jpt Innovative Peptide Solutions (Berlin, Germany). The peptide was added to the respective membrane preparation and the solution was incubated with the myc-antibody-coupled magnetic beads for 30 min at room temperature. The co-IPs containing the peptide-free membrane preparations were processed under identical conditions. For the native protease cleavage assay, membrane preparations^36^ of transfected HEK293T cells were incubated for 5 h at room temperature (RT) and processed for western blotting. No protease inhibitors were added to the membrane preparations prior to the incubation. For immunoblotting, antibodies were used in following dilutions: mouse anti-myc (see above), 1:2000; mouse anti-GFP (Clontec, Takara Bio Inc., Shiga, Japan), 1:2000; mouse anti-ATPase (anti alpha3, ab2826, Abcam, Cambridge, UK), 1:2000.

### Sucrose density gradient centrifugation

For sucrose density gradient centrifugation, HEK293T cells were transiently transfected with the respective peripherin-2 constructs. Cells were harvested 48 h post transfection and homogenized in a lysis buffer comprising 0.5% Triton X-100, 150 mM NaCl and 2 mM CaCl_2_ using the Potter S homogenizer (see above). Continuous density gradients of 5–20% (wt/vol) sucrose were prepared by underlayering 0.5 ml of 5%, 10%, 15% and 20% sucrose containing 0.1% Triton X-100 and 10 mM *N*-ethylmaleimide (Sigma-Aldrich, St. Louis, MO, USA). The gradient was left at RT for 1 h to facilitate diffusion and chilled on ice for 30 min prior to sample application. To determine the weight of single peripherin-2 and Rom-1 protein complexes in each immunoblot, DNA standards of defined molecular weights (75 bp–20.000 bp, i.e. 49 kDa–13,000 kDa, GeneRuler 1 kb Plus (Thermo Fisher Scientific, Waltham, MA, USA)) were mixed to lysates containing 200 µg of the respective proteins. The mixture was carefully layered on top of the gradient and centrifuged at 46,700 rpm for 2 h at 4 °C in a Beckman Coulter Optima L-80K Ultracentrifuge (Beckman Coulter Biomedical GmbH, Munich, Germany). Afterwards, the centrifuge tube was punctured and fractions were collected dropwise (5 drops/tube). Fractions from each sucrose gradient were separately used for immunoblotting with myc- or flag-specific antibodies and for agarose gel electrophoresis to detect the DNA standards. The suitability of DNA standards was first validated for per^WT^ only. As shown in Fig. 3a, per^WT^ gives rise to well-defined fractions of non-covalent tetramers, covalent octamers, and high-order oligomers. The molecular weight of the DNA in the same fractions very nicely correlated with the molecular weights of the corresponding per^WT^ complexes. Therefore, for each of the remaining immunoblots, DNA standards were used to determine the fractions containing the different per^WT^, per^MT^, or Rom-1 complexes.

### Photometric FRET Measurements

3 cube FRET was performed as described in detail in Butz *et al*.^37^ with modifications published in Becirovic *et al*. and Nguyen *et al*.^18, 19^. Briefly, transfected HEK293T cells and isolated rod OS were measured in a FRET imaging solution comprising 2 mM CaCl_2_, 10 mM glucose, 10 mM HEPES sodium salt, 5 mM KCl, 1 mM MgCl_2_, 140 mM NaCl, pH 7.4, at RT. 3 cube FRET measurements were conducted using a Leica DMI6000B inverted fluorescent microscope (Leica, Wetzlar, Germany) in combination with a photomultiplier detection system including a photomultiplier tube (Horiba, London, Ontario, Canada). As excitation source, a DeltaRamX monochromator was used and FRET data were acquired with FelixGX software (Horiba, London, Ontario, Canada). FRET filter cubes were as follows (excitation, dichroic mirror, emission): cerulean/CFP (ET436/20 × ; T455lp; ET480/40m), FRET (ET436/20 × ; T455lp; ET535/30m) and citrine/YFP (ET500/20 × ; T515lp; ET535/30m) (Chroma technology, Vermont, USA). The fluorescence intensities were measured in single cells co-expressing varying, but sufficient levels of cerulean- and citrine-tagged proteins with the respective filter cube. Subsequent FRET ratios (FR) were calculated from the signal intensities according to the 3 cube FRET equation described in Shaltiel *et al*.^38^. Apparent FRET efficiencies (E_A_) at given cerulean/citrine molar ratios (MR) were obtained from equation (1):

1 $${{\rm{E}}}_{{\rm{A}}}\,=\,[\mathrm{FR}-1]\cdot \frac{{{\rm{\varepsilon }}}_{{\rm{citrine}}}\,(\mathrm{436})}{{{\rm{\varepsilon }}}_{{\rm{cerulean}}}\,(\mathrm{436})}$$

ε_citrine_ and ε_cerulean_ represent the setup specific average molar extinction coefficients for the fluorophores citrine or cerulean, respectively. FRET data were analyzed using FelixGX software (see above) and Excel (Microsoft Corporation, Redmond, Washington, USA).

Binding curves were calculated using equation (2):

2 $${{\rm{E}}}_{{\rm{A}}}=\frac{{{\rm{E}}}_{{\rm{Amax}}}\cdot {\rm{MR}}}{{\rm{K}}\,+\,\mathrm{MR}}$$

E_Amax_ is the maximal FRET efficiency that can be calculated for saturated donor concentrations and K is an analogon of the dissociation constant. *Ex vivo* FRET experiments were conducted on single rod OS isolated from six pooled retinas for each FRET pair.

### rAAV preparation and subretinal injections

The production of single-strand AAVs (rAAV 2/8 YF) containing the hRHO promoter and the subretinal injection technique were described previously^30^. 10^10^ rAAV particles were delivered subretinally to wild type C57Bl6/J mice by a single injection on postnatal day 14. Four weeks post injection, all injected retinas were analyzed for fluorescence using scanning laser ophthalmoscopy (Spectralis, Heidelberg Engineering, Dossenheim, Germany).

### Preparation of outer segments

A detailed procedure of rod OS preparation was described recently^17, 19^. Briefly, mice were sacrificed four weeks post injection and the isolated retinas were collected in a 1.5-ml reaction tube containing 100 µl of PBS. The OS were separated from the retina by vortexing for 15–30 s and centrifuging for 30 s at 500 × g. Afterwards the OS containing supernatant was collected and used for subsequent FRET measurements.

### Immunohistology and Confocal Microscopy

For immunohistology, wild type C57BL6/J mice were injected on P14 with the respective rAAV particles. Four weeks post injection, the retinas were dissected and processed for immunohistology as described^39^. Retinal and OS images were obtained by the TCS SP8 confocal scan microscope (Leica, Wetzlar, Germany), acquired with the LASX software (Leica, Wetzlar, Germany), and processed with the ImageJ software (National Institutes of Health, Bethesda, MD, USA). We used the rabbit anti-CNGB1a antibody (1:10.000^40^) as marker for rod outer segments. Citrine was excited with the 514 nm laser and detected with the Hybrid (HyD) detector using a 520–540 nm emission filter. Cerulean was excited using the 448 nm laser and detected via the HyD detector with a 460–480 nm emission filter. Images were taken using the 40x and 63x objectives.

### Confocal scanning laser ophthalmoscopy and optical coherence tomography

Confocal scanning laser ophthalmoscopy (cSLO) and optical coherence tomography (OCT) were performed 6 months post injection using a modified Spectralis HRA + OCT system (Heidelberg Engineering, Dossenheim, Germany) as described recently^41^. Fluorescence signals were examined at 488 nm excitation for citrine/cerulean detection (BP 550/49 nm emission filter).

### Statistics

All values are given as mean ± SEM, and *n* is the number of trials. For multiple comparisons one-way ANOVA followed by the Tukey’s test was used.

### Data availability

All data generated or analyzed during this study are included in this published article (and its Supplementary Information files).

## Electronic supplementary material

Supplementary Information
