# Supplementary material for: Peripherin-2 and Rom-1 have opposing effects on rod outer segment targeting of retinitis pigmentosa-linked peripherin-2 mutants
Source: Sci Rep. 2017 May 24;7:2321. doi: 10.1038/s41598-017-02514-5 (PMC5443838; doi:10.1038/s41598-017-02514-5)
Supplement: Supplementary file 1 — Supplementary Information [file 41598_2017_2514_MOESM1_ESM.pdf]

Peripherin-2 and Rom-1 have opposing effects on rod outer segment targeting of retinitis pigmentosa-linked peripherin-2 mutants

Sybille Böhm[1,2#], Lisa M. Riedmayr[1,2#], O.N. Phuong Nguyen[1,2], Andreas Gießl[3], Toni Liebscher[1,2], Elisabeth S. Butz[1,2], Christian Schön[1,2], Stylianos Michalakis[1,2], Christian Wahl-Schott[1,2], Martin Biel[1,2] and Elvir Becirovic[1,2*]

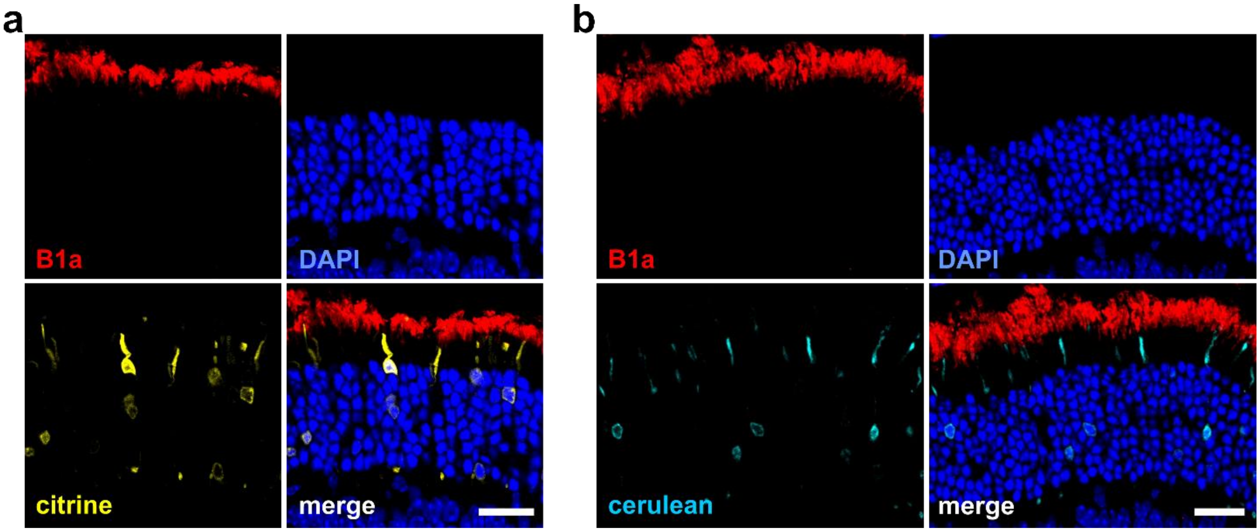

Supplementary Figure 1: Expression profile of citrine and cerulean in transduced photoreceptors. Scale bar, 30 µm.

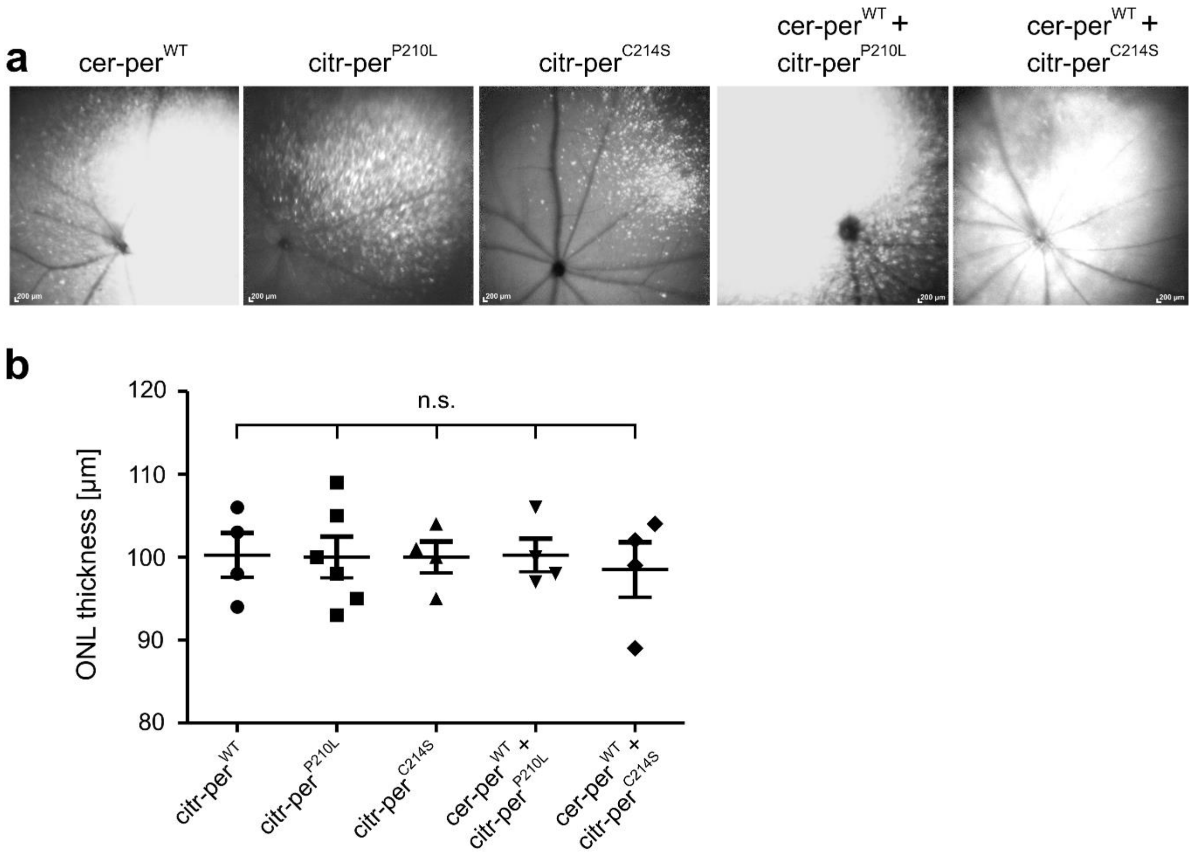

Supplementary Figure 2: Long-term effects of per^{WT} and per^{MT} expression on retinal degeneration in transduced murine photoreceptors. A, Representative confocal scanning laser ophthalmoscopy (cSLO) of mice injected with the single constructs as indicated. Note that, in contrast to the confocal microscope settings, citrine and cerulean fluorescence (white signal) cannot be distinguished using cSLO. B, measurements of the outer nuclear layer (ONL) thickness of injected animals using optical coherence tomography. The single values are as follows: citr-per^{WT} (n=4) 100.3 ± 2.7 µm; citr-per^{P210L} (n=6) 100.0 ± 2.5 µm; citr-per^{C214S} (n=4) 100.0 ± 1.9 µm; citr-per^{WT} + citr-per^{P210L} (n=4) 100.3 ± 2.0 µm; citr-per^{WT} + citr-per^{C214S} (n=4) 98.5 ± 3.3 µm. Statistical analysis was done with one-way ANOVA followed by the Tukey's test for multiple comparisons. n.s., not significant.

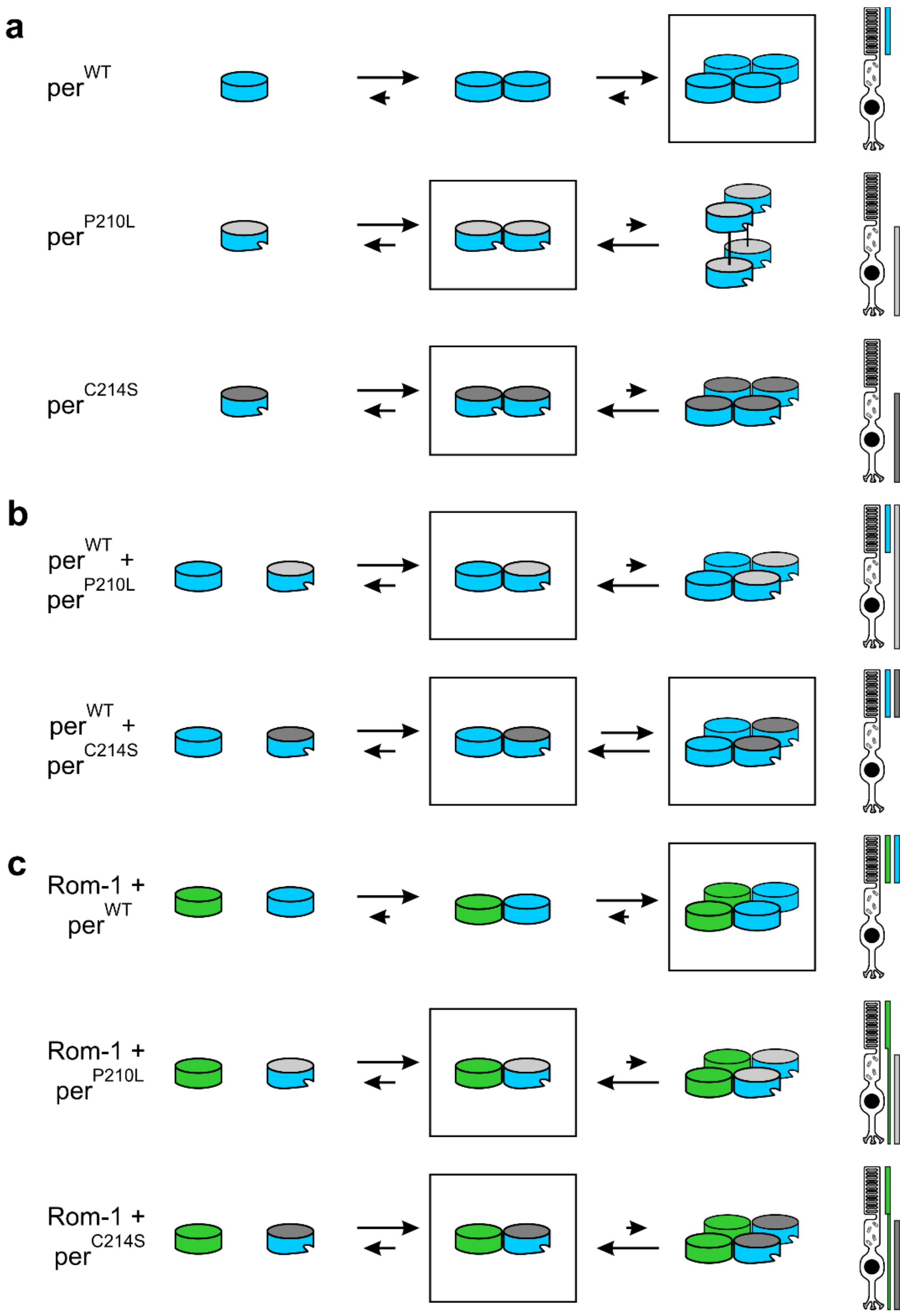

Supplementary Figure 3: Tentative model of per^{MT} protein assembly and rod OS targeting in presence or absence of transgenic per^{WT} and Rom-1. For reasons of clarity, octamers, higher-order oligomers, and aggregates are not included in this model. The rectangle highlights the major SDGC fraction(s) for the single homo- or heterogenic interactions and the arrow length correlates with the preferential direction within the equilibrium. The misfolding of the distal part of the D2 loop caused by both mutants is symbolized by an incision. The localization of the respective constructs in rod photoreceptors (right panel) is indicated by colored bars symbolizing color-coded per^{WT}, per^{MT}, or Rom-1 constructs. The bar thickness represents the estimated relative amount of the detected constructs in the respective compartment.

Supplementary Table 1: FRET measurements from HEK293T cells and from isolated rod OS.

	HEK293T								rod OS		
	WT-WT	WT-P210L	WT-C214S	P210L-P210L	C214S-C214S	WT-ROM1	ROM-1-P210L	ROM-1-C214S	WT-WT	WT-P210L	WT-C214S
E_{Amax}	34.2	9.44	8.58	30.7	26.47	15.64	6.58	6.32	25.96	37.12	26.47
% of WT-WT or WT-ROM-1		28	25	90	77		42	40		143	102
n	53	53	50	53	54	48	48	48	45	32	44

Supplementary Table 2: Quantification of the SDGC experiments. Values are given as mean values ± SEM.

			Immunoreactivity (% of Total)										
			Fraction										
			1	2	3	4	5	6	7	8	9	10	11
n = 3	**WT**	Non-covalent	0.00	0.00	0.00	2.78 ± 2.78	9.33 ± 0.71	11.53 ± 1.38	11.70 ± 2.15	10.98 ± 2.06	2.65 ± 2.65	2.76 ± 2.76	1.68 ± 1.68
		Disulfide bond	0.00	2.05 ± 2.05	4.73 ± 2.39	7.97 ± 1.60	11.78 ± 1.26	12.46 ± 1.50	7.61 ± 0.57	0.00	0.00	0.00	0.00
n = 4	**P210L**	Non-covalent	0.00	0.00	0.00	0.00	0.00	0.00	0.00	0.00	2.10 ± 2.10	20.35 ± 4.66	19.00 ± 6.53
		Disulfide bond	0.00	0.00	0.00	0.00	2.86 ± 2.86	5.44 ± 3.16	4.81 ± 2.79	5.78 ± 2.13	2.67 ± 2.67	1.78 ± 1.78	0.00
		Aggregate	0.00	0.00	2.08 ± 2.08	8.28 ± 3.17	10.83 ± 1.53	8.80 ± 3.07	3.18 ± 2.67	2.02 ± 1.78	0.00	0.00	0.00
n = 3	**C214S**	Non-covalent	0.00	0.00	0.00	0.00	2.61 ± 2.61	4.36 ± 4.36	3.42 ± 3.42	3.34 ± 3.34	18.23 ± 13.07	27.29 ± 14.59	5.20 ± 5.20
		Aggregate	7.28 ± 3.77	7.65 ± 4.20	5.27 ± 5.27	4.03 ± 4.03	6.78 ± 6.78	4.53 ± 4.53	0.00	0.00	0.00	0.00	0.00
n = 5	**P210L + WT IB: anti-myc**	Non-covalent	0.00	1.74 ± 1.74	2.84 ± 2.84	3.13 ± 3.13	5.13 ± 3.15	4.33 ± 2.80	2.07 ± 2.07	4.45 ± 2.73	23.39 ± 4.14	27.42 ± 5.71	12.38 ± 5.53
		Disulfide bond	0.00	0.00	0.00	0.00	0.00	0.00	0.00	0.00	0.00	0.00	0.00
		Aggregate	0.00	1.36 ± 1.36	0.00	2.01 ± 1.27	3.57 ± 2.37	3.94 ± 2.49	2.23 ± 2.23	0.00	0.00	0.00	0.00
	**P210L + WT IB: anti-flag**	Non-covalent	0.00	0.00	0.00	0.00	2.37 ± 2.37	1.85 ± 1.85	9.13 ± 1.95	11.07 ± 4.26	10.79 ± 5.76	11.68 ± 3.67	0.00
		Disulfide bond	3.68 ± 3.68	4.67 ± 4.67	6.60 ± 3.45	7.86 ± 4.13	12.61 ± 0.59	11.95 ± 2.62	3.35 ± 3.35	2.40 ± 2.40	0.00	0.00	0.00
n = 4	**C214S + WT IB: anti-myc**	Non-covalent	0.00	0.00	0.00	0.00	4.45 ± 2.64	5.22 ± 3.09	5.91 ± 3.74	16.61 ± 3.49	12.33 ± 4.41	20.32 ± 4.78	19.65 ± 10.11
		Aggregate	0.00	0.00	5.87 ± 3.60	7.81 ± 2.74	1.82 ± 1.82	0.00	0.00	0.00	0.00	0.00	0.00
	**C214S + WT IB: anti-flag**	Non-covalent	0.00	0.00	0.00	0.00	1.45 ± 1.45	1.67 ± 1.12	5.95 ± 1.39	3.40 ± 1.90	6.41 ± 0.57	7.52 ± 0.92	4.11 ± 4.11
		Disulfide bond	5.51 ± 2.76	8.75 ± 1.12	10.40 ± 0.57	11.06 ± 0.38	11.38 ± 0.63	9.58 ± 0.63	7.24 ± 0.64	3.46 ± 1.90	2.11 ± 2.11	0.00	0.00
n = 3	**WT + Rom-1 IB: anti-myc**	Non-covalent	0.00	0.00	0.00	0.00	8.22 ± 0.86	10.20 ± 1.46	6.34 ± 3.17	4.70 ± 4.70	2.77 ± 2.77	0.00	0.00
		Disulfide bond	8.48 ± 4.42	10.61 ± 1.81	10.18 ± 1.60	9.69 ± 1.87	10.50 ± 2.20	10.41 ± 1.38	7.92 ± 0.20	0.00	0.00	0.00	0.00
	**WT + Rom-1 IB: anti-flag**	Non-covalent	0.00	0.00	0.00	1.45 ± 1.45	5.40 ± 2.89	14.96 ± 2.60	17.61 ± 1.74	18.26 ± 9.93	19.35 ± 3.61	8.99 ± 5.91	3.61 ± 3.61
		Disulfide bond	0.00	0.00	0.00	0.00	3.49 ± 1.85	4.55 ± 2.65	2.33 ± 2.33	0.00	0.00	0.00	0.00
n = 3	**P210L + Rom-1 IB: anti-myc**	Non-covalent	0.00	0.00	0.00	0.00	0.00	0.00	0.00	4.56 ± 4.56	22.76 ± 2.11	31.48 ± 6.45	28.67 ± 9.40
		Disulfide bond	0.00	0.00	0.00	0.00	0.00	0.00	0.00	0.00	0.00	0.00	0.00
		Aggregate	0.00	5.60 ± 5.60	0.00	0.00	5.01 ± 5.01	1.93 ± 1.93	0.00	0.00	0.00	0.00	0.00
	**P210L + Rom-1 IB: anti-flag**	Non-covalent	0.00	0.00	0.00	0.00	0.00	0.00	0.00	0.00	19.68 ± 9.84	43.50 ± 2.28	36.82 ± 12.12
		Disulfide bond	0.00	0.00	0.00	0.00	0.00	0.00	0.00	0.00	0.00	0.00	0.00
n = 3	**C214S + Rom-1 IB: anti-myc**	Non-covalent	0.00	0.00	0.00	0.00	0.00	4.59 ± 4.59	4.91 ± 4.91	0.00	29.00 ± 2.51	31.53 ± 3.67	24.34 ± 3.41
		Aggregate	0.00	0.00	0.00	0.00	5.64 ± 5.64	0.00	0.00	0.00	0.00	0.00	0.00
	**C214S + Rom-1 IB: anti-flag**	Non-covalent	0.00	0.00	0.00	0.00	0.00	0.00	0.00	2.43 ± 2.43	28.57 ± 6.38	26.46 ± 2.94	21.20 ± 2.41
		Disulfide bond	0.00	0.00	0.00	0.00	0.00	0.00	1.75 ± 1.75	6.79 ± 4.15	10.18 ± 0.14	2.60 ± 2.60	0.00
